# Discordance between the triglyceride glucose index and HOMA-IR in incident albuminuria: a cohort study from China

**DOI:** 10.1186/s12944-021-01602-w

**Published:** 2021-12-05

**Authors:** Wei Gao, Jialu Wang, Yan Chen, Hongmei Qiao, Xiaozhong Qian, Zhuojun Xin, Zhiyun Zhao, Tiange Wang, Yu Xu, Min Xu, Yufang Bi, Mian Li, Jinli Gao

**Affiliations:** 1 Songnan Town Community health Service Center, Baoshan District, Shanghai, China; 2 Department of Endocrine and Metabolic Diseases, Shanghai Institute of Endocrine and Metabolic Diseases, Ruijin Hospital, Shanghai Jiaotong University School of Medicine, 197 Rui-Jin 2nd Road, Shanghai, 200025 China; 3 Shanghai National Clinical Research Center for Metabolic Diseases, Key Laboratory for Endocrine and Metabolic Diseases of the National Health Commission of the PR China, Shanghai National Center for Translational Medicine, Ruijin Hospital, Shanghai Jiaotong University School of Medicine, 197 Rui-Jin 2nd Road, Shanghai, 200025 China

**Keywords:** Chronic kidney disease, Discordance analysis, Insulin resistance, Triglyceride-glucose index

## Abstract

**Background:**

To date, there have no study comparing the associations between TyG index and HOMA-IR on the risk of incident albuminuria. Accordingly, the objective of the present study is to use discordance analysis to evaluate the diverse associations between TyG index and HOMA-IR on the risk of incident albuminuria.

**Methods:**

A community-based prospective cohort study was performed with 2446 Chinese adults. We categorized participants into 4 concordance or discordance groups. Discordance was defined as a TyG index equal to or greater than the upper quartile and HOMA-IR less than the upper quartile, or vice versa.

**Results:**

During a median follow-up period of 3.9 years, 203 of 2446 participants developed incident albuminuria (8.3%). In the multivariable logistic analyses, the high TyG index tertile group was associated with a 1.71-fold (95% confidence interval (CI) 1.07–2.72) higher risk of incident albuminuria, comparing with the low tertile group. Participants in TyG (+) & HOMA-IR (−) group had a greater risk of incident albuminuria compared with those in TyG (−) & HOMA-IR (−) group after multivariate adjustment. Subgroup analyses showed that low HOMA-IR and discordantly high TyG index was closely related to a highest risk of incident albuminuria in cardiovascular metabolic disorder subjects.

**Conclusions:**

Participants with a discordantly high TyG index had a significantly greater risk of incident albuminuria, especially in metabolic dysfunction subjects. The TyG index might be a better predictor of early stage of chronic kidney disease than HOMA-IR for subjects with metabolic abnormality.

**Supplementary Information:**

The online version contains supplementary material available at 10.1186/s12944-021-01602-w.

## Background

Chronic kidney disease (CKD), a major disease burden, affects 8 to 16% of the population worldwide [1, 2]. Globally, metabolic disorders are the most common reasons for CKD [3]. Since extensive evidence has confirmed the strong association between CKD and an increased risk of cardiovascular disease [4, 5]. Dysregulated metabolic factors, including diabetes mellitus, hypertension and dyslipidaemia, play leading roles in mediating this relationship, the early identification of CKD is critical for preventing clinical cardiovascular events. Additionally, large-scale studies have proven that albuminuria is a sensitive biological marker of progression of kidney diseases in early stage of CKD [6], and increased urinary albumin excretion is also an important indicator of cardiovascular metabolic risk factors [7, 8].

Insulin resistance (IR) is an early metabolic change in individuals with CKD [9]. Since the hyperinsulinaemic-euglycaemic clamp (HIEC) is a well-accepted ‘gold standard’ approach for evaluating IR, the homeostasis model assessment of IR (HOMA-IR) is a relatively most widely used tool to assess IR. Moreover, considering the convenience of implementation, researchers often use the upper quartile of HOMA-IR as the standard in population research. With regard to triglycerides (TGs) and high-density lipoprotein (HDL) cholesterol are components of metabolic disorders [10]. Previous studies have reported that lipid ratios, such as TG/HDL cholesterol, the non-HDL cholesterol/HDL cholesterol and triglyceride-glucose (TyG) index, are good indicators of the early identification of IR and have been widely used in clinical practice [11]. Moreover, the TyG index, calculated by fasting glucose and triglycerides, has been shown to perform better than HOMA-IR [12] and to be significantly correlated with HIEC [13].

To date, this kind of studies have rare explored the connection of the TyG index and albuminuria. A community-based study designed by Zhao et al. [14] recently showed that, in elderly individuals, higher levels of the TyG index were closely related to a greater risk of CKD and microalbuminuria. However, no studies have compared the abilities of the TyG index and HOMA-IR to measure new-onset albuminuria in the general population. Therefore, the present study was carried out to use discordance analysis for the assessment and further comparison of the effects of TyG index and HOMA-IR on incident albuminuria risk.

## Methods

### Study design and participants

**Fig. 1:**
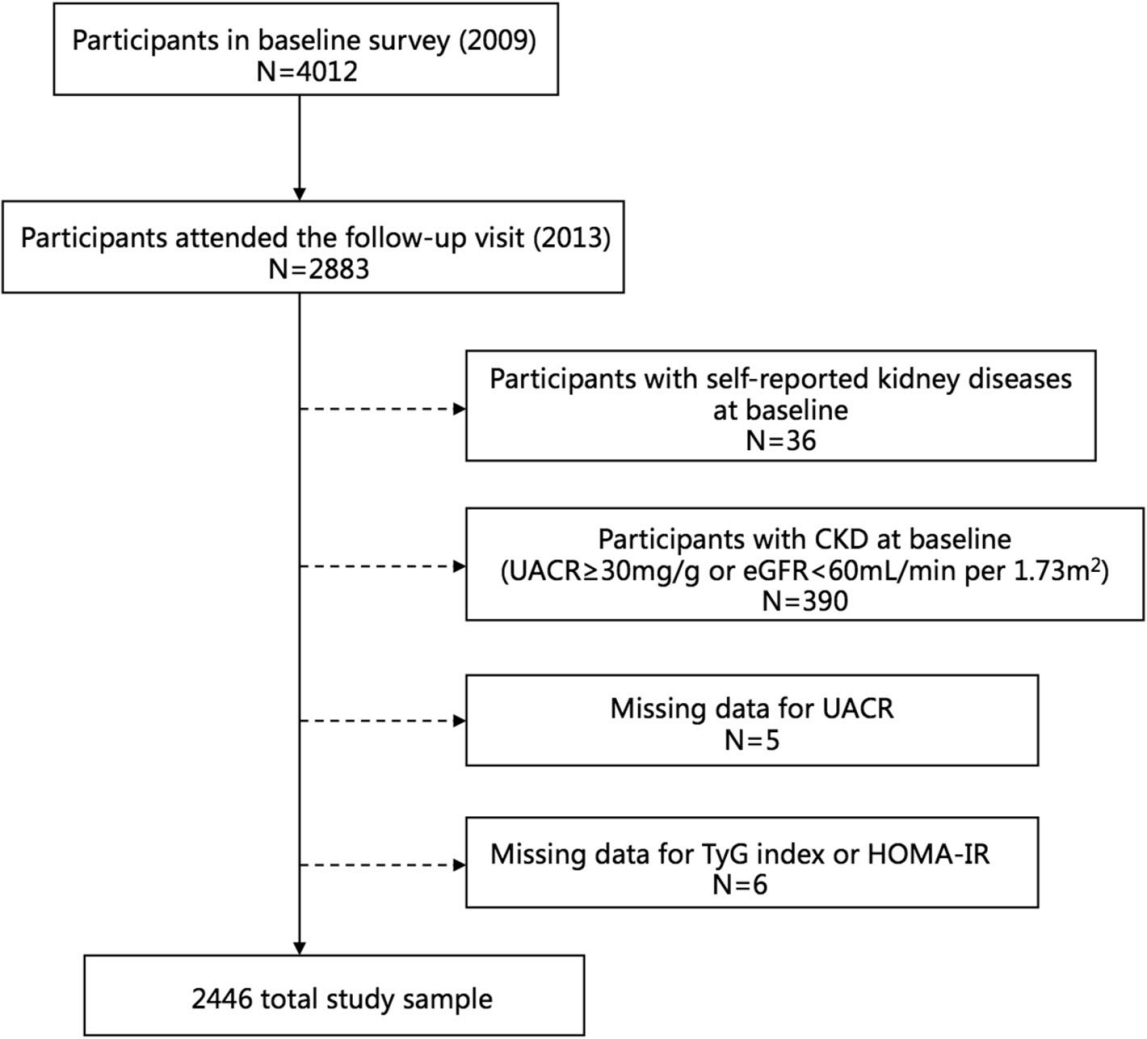
Participant Flow Diagram of the Study. TyG, triglyceride glucose; HOMA-IR, homeostasis model assessment for insulin resistance; CKD, chronic kidney disease; UACR, urinary albumin-to-creatinine ratio; eGFR: estimated glomerular filtration rate

A community-based investigation was undertaken from June to August 2009 in the Songnan community, Baoshan District, Shanghai, China. A circumstantial introduction of this research population has been previously published [15, 16]. In total, 4012 participants underwent this examination at baseline. Serum and urine specimens were collected to detect TyG and urinary albumin-to-creatinine ratio (UACR). All participants were asked for to take part in a follow-up visit, of which 2883 individuals attended and tested for UACR between March and May in 2013. This current research was designed to explore the relationship of the TyG index and HOMA-IR to new-onset albuminuria. The exclusion criteria for the current analysis were subjects who (a) had self-reported kidney diseases at baseline (*n* = 36); (b) had UACR ≥ 30 mg/g or estimated glomerular filtration rate (eGFR) < 60 mL/min per 1.73 m^2^ (*n* = 390); (c) lacked UACR (*n* = 5); and (d) had missing data for TyG index or HOMA-IR (*n* = 6). Finally, 2446 individuals were included in this current study (Fig. 1).

The upper quartiles of baseline for the TyG index and HOMA-IR were calculated to classify participants into following 2 classes: low (lower than the upper quartiles) and high (equal to or higher than the upper quartiles). Then, participants were divided into 4 groups on the basis of the low/high value of the TyG index and HOMA-IR, as follows: TyG (−) & HOMA-IR (−), TyG (+) & HOMA-IR (−), TyG (−) & HOMA-IR (+) and TyG (+) & HOMA-IR (+) groups. Discordance groups were presented as the TyG (+) & HOMA-IR (−) and TyG (−) & HOMA-IR (+) groups.

The study protocol was approved by the Institutional Review Board of Rui Jin Hospital, Shanghai Jiao Tong University School of Medicine. All participants have written informed consent.

### Data collection and measurements

The trained physicians used a normalized questionnaire to collect information including sociodemographic features, education levels, lifestyle and history of chronic disease with two face-to-face interviews. The status of current smoking or drinking was defined as smoking or drinking frequently in the past half year. The International Physical Activity Questionnaire was often used to evaluate the degrees of physical activity [17]. Body weight, height and blood pressure (BP) were measured by experienced nurses on the basis of a standard protocol [15]. Participants were asked to rest for 5 min, and their seated blood pressure was measured three times on a nondominant arm with a 1 min interval. The average value of blood pressure was applied in the following analysis. Pulse pressure (PP) was obtained as the mean of three measurements by subtracting diastolic BP (DBP) from systolic BP (SBP).

Since the detection methods and instruments of blood samples and first-voided urine samples at early morning were previously described in published studies, repeat specification was no longer required here [15, 18, 19]. The TyG index was obtained by the formula: ln (fasting triglycerides (mg/dL) × fasting glucose (mg/dL)/2) [13]. The HOMA-IR index was obtained as fasting insulin (IU/mL) × fasting glucose (mmol/L)/22.5 [20].

### Definitions

New-onset albuminuria was regarded as a UACR level of 30 mg/g or higher. The definition of CKD was an eGFR ≤ 60 mL/min per 1.73 m^2^ or albuminuria. Hypertension was accepted as a SBP level of 140 mmHg or higher, DBP level of 90 mmHg or higher, or self-reported previous history of hypertension by professionals. The definition of diabetes was a fasting plasma glucose (FPG) level of 7.0 mmol/L or higher, 2-h glucose after 75-g oral glucose tolerance test (OGTT) level of 11.1 mmol/L or higher, glycated haemoglobin (HbA1c) level of 6.5% or higher, or self-reported diagnosis by physicians and taking hypoglycaemic medications on the basis of the 2010 American Diabetes Association (ADA) criteria.

### Statistical analysis

All data were analysed on the SAS version 9.4 platform (SAS Institute, Cary, NC, USA). A two-tailed *P* value < 0.05 was considered statistically significant. Baseline variables were compared according to 4 concordance or discordance groups. Continuous data were shown as means ± standard deviation, while categorical variables were displayed as numbers (%). Differences in baseline characteristics among the 4 concordance or discordance groups were carried out by one-way analysis of variance or the χ2 test.

The relationships of the TyG index tertiles, HOMA-IR tertiles and the 4 concordance or discordance groups with new-onset albuminuria were explored using multivariate-adjusted logistic regression models. Covariates involved in the analysis included age, sex, status of current smoking or drinking, education levels, physical activity, HbA1c, PP, HDL cholesterol, LDL cholesterol, total cholesterol, BMI and medication usage of angiotensin-converting enzyme inhibitors (ACEIs) or angiotensin receptor blockers (ARBs).

Furthermore, stratified analysis of the 4 concordance or discordance groups with the risk of new-onset albuminuria was repeated according to the status of diabetes, hypertension and age categories (≥ 60 years old or < 60 years old).

Additionally, the above analyses were repeated on the outcome of incident CKD. The flow diagram was shown in Supplementary Fig. 1, and the results were also shown in the supplementary materials.

## Results

### Baseline characteristics of participants in 4 concordance or discordance groups according to the TyG index and HOMA-IR

Baseline demographic and clinical characteristics were compared across the 4 concordance or discordance groups according to low or high categories for the TyG index and HOMA-IR (Table 1). The average age of enrolled subjects was 59.17 years old, and 968 of them were men (39.6%). Age, current smoking status, education level, diabetes, hypertension, and dyslipidaemia were different among the 4 groups. Furthermore, participants in TyG (−) & HOMA-IR (+) group were more probable to get a higher body mass index (BMI), SBP, DBP, FPG, fasting insulin, post load glucose and LDL cholesterol in comparison with TyG (+) & HOMA-IR (−) group and had a higher prevalence of diabetes and hypertension. Scatterplots and prevalence of discordance and concordance defined according to the upper quartile values of TyG index and HOMA-IR were depicted in Supplementary Fig. 2.

**Table 1:** Baseline characteristics of the concordance and discordance according to low or high TyG index and HOMA-IR categories

	Total	TyG(−)&HOMA-IR(−)	TyG(+)&HOMA-IR(−)	TyG(−)&HOMA-IR(+)	TyG(+)&HOMA-IR(+)	*P* value
N	2446	1530	304	305	307	
Age (year)	59.17 ± 8.94	58.64 ± 9.04	59.58 ± 8.62	60.64 ± 9.09	59.92 ± 8.40	0.0004
Male, n(%)	968 (39.6)	599 (39.2)	148 (48.7)	102 (33.4)	119 (38.8)	0.51
Current smoking, n(%)	488 (20.0)	311 (20.3)	84 (27.6)	40 (13.1)	53 (17.3)	0.05
Current drinking, n(%)	443 (18.1)	268 (17.5)	73 (24.0)	54 (17.7)	48 (15.6)	0.72
Physical activity (MET-h/wk)	23.10 (11.55–46.20)	23.10 (11.55–46.20)	23.10 (11.55–46.20)	23.10 (11.55–46.20)	23.10 (11.55–37.10)	0.005
Highschool education, n(%)	1949 (79.7)	1255 (82.0)	242 (79.6)	222 (72.8)	230 (74.9)	< 0.0001
SBP (mmHg)	135.98 ± 20.19	132.82 ± 19.91	138.04 ± 19.29	141.57 ± 19.78	143.99 ± 19.23	< 0.0001
DBP (mmHg)	78.36 ± 9.79	76.84 ± 9.70	79.64 ± 9.59	80.71 ± 9.27	82.23 ± 9.24	< 0.0001
PP (mmHg)	57.63 ± 16.38	55.98 ± 16.00	58.40 ± 15.63	60.86 ± 17.07	61.76 ± 17.00	< 0.0001
Height (cm)	159.93 ± 7.92	159.92 ± 7.75	160.53 ± 8.09	159.30 ± 7.81	159.99 ± 8.67	0.74
Weight (kg)	64.49 ± 10.59	62.17 ± 9.62	65.14 ± 9.58	69.92 ± 11.12	69.93 ± 11.58	< 0.0001
BMI (kg/m^2^)	25.17 ± 3.50	24.28 ± 3.19	25.20 ± 2.85	27.50 ± 3.54	27.29 ± 3.57	< 0.0001
FPG (mmol/L)	5.13 (4.70–5.82)	4.90 (4.60–5.30)	5.50 (5.00–6.20)	5.60 (5.00–6.50)	6.90 (5.70–9.00)	< 0.0001
Fasting insulin (μIU/ml)	7.00 (4.57–10.26)	5.57 (3.85–7.59)	6.81 (4.97–8.54)	14.04 (12.09–17.03)	12.95 (10.57–17.33)	< 0.0001
Post load glucose (mmol/L)	7.60 (6.10–11.30)	6.90 (5.60–8.40)	8.80 (7.00–13.20)	9.10 (7.00–14.55)	14.70 (10.70–19.25)	< 0.0001
HbA1c (%)	6.10 (5.80–6.60)	5.90 (5.70–6.20)	6.30 (5.80–6.90)	6.40 (6.00–7.10)	7.40 (6.50–8.90)	< 0.0001
HOMA-IR	1.65 (1.03–2.63)	1.25 (0.82–1.73)	1.79 (1.32–2.22)	3.45 (2.92–4.34)	4.01 (3.18–5.73)	< 0.0001
Total cholesterol (mmol/L)	5.15 ± 0.96	5.03 ± 0.89	5.42 ± 1.03	5.01 ± 0.98	5.63 ± 1.04	< 0.0001
Triglyceride (mmol/L)	1.41 (0.97–2.07)	1.16 (0.86–1.54)	2.89 (2.44–3.62)	1.35 (1.00–1.68)	2.65 (2.11–3.43)	< 0.0001
HDL cholesterol (mmol/L)	1.36 ± 0.31	1.42 ± 0.32	1.26 ± 0.25	1.29 ± 0.29	1.24 ± 0.20	< 0.0001
LDL cholesterol (mmol/L)	2.39 ± 0.68	2.35 ± 0.63	2.29 ± 0.74	2.53 ± 0.66	2.52 ± 0.80	< 0.0001
UACR (mg/g)	4.89 (2.45–9.23)	4.58 (2.32–8.40)	4.41 (2.42–9.47)	6.02 (2.92–11.92)	6.24 (2.93–12.13)	< 0.0001
eGFR (ml/min/1.73m^2^)	90.90 ± 12.75	91.25 ± 12.58	90.13 ± 12.90	89.66 ± 12.96	91.22 ± 13.20	0.31
TyG index	8.74 ± 0.68	8.40 ± 0.45	9.53 ± 0.32	8.68 ± 0.41	9.72 ± 0.52	< 0.0001
Medication use of ACEIs or ARBs, n(%)	74 (3.0)	33 (2.2)	8 (2.6)	13 (4.3)	20 (6.5)	< 0.0001
Diabetes, n(%)	860 (35.2)	310 (20.5)	138 (46.0)	167 (54.8)	245 (80.1)	< 0.0001
Hypertension, n(%)	1281 (52.4)	674 (45.1)	186 (61.6)	199 (66.3)	222 (72.6)	< 0.0001
History of MACEs, n(%)	213 (8.7)	112 (7.3)	37 (12.2)	28 (9.2)	36 (11.7)	0.007
MACEs at follow-up visit, n(%)	160 (6.5)	89 (5.8)	29 (9.5)	18 (5.9)	24 (7.8)	0.20

Data are expressed as mean ± standard deviation, median (interquartile range) or as n (%). MET, metabolic equivalent task; BMI, body mass index; SBP, systolic blood pressure; DBP, diastolic blood pressure; PP, pulse pressure; FPG, fasting plasma glucose; HbA1c, glycated hemoglobin; LDL, low density lipoprotein; HDL, high density lipoprotein; UACR, urinary albumin-to-creatinine ratio; eGFR, estimated glomerular filtration rate; TyG, triglyceride-glucose; HOMA-IR, homeostasis model assessment of insulin resistance; ACEIs, angiotensin-converting enzyme inhibitors; ARBs, angiotensin receptor blockers

### Relationship of the TyG index, HOMA-IR and concordance or discordance groups with new-onset albuminuria and CKD

Table 2 shows the odds ratios (ORs) of new-onset albuminuria in participants according to TyG index tertiles, HOMA-IR tertiles and 4 concordance or discordance groups. The prevalence rates of increased new-onset albuminuria were 5.4, 7.1, and 12.4% from the range of TyG index tertiles low to high, and 4.4, 8.1, and 12.4% of HOMA-IR tertiles, respectively. Compared with the low tertile, the high TyG index tertile (OR: 1.71; 95% CI 1.07–2.72) and the high HOMA-IR tertile (OR: 1.72, 95% CI 1.07–2.78) had a greater risk of developing new-onset albuminuria. Considering the discordance analysis, participants in TyG (+) & HOMA-IR (−) group had a higher risk of new-onset albuminuria than those in TyG (−) & HOMA-IR (−) group after adjustment of all covariates (OR: 1.69, 95% CI 1.04–2.75). However, there was no significant result in the risk of new-onset albuminuria in participants with low TyG but high HOMA-IR.

**Table 2:** Incidence of albuminuria using the TyG index, HOMA-IR and concordance/discordance groups

		albuminuria		
	Cases/participants (%)	Model 1	Model 2	Model 3
		OR (95% CI)	OR (95% CI)	OR (95% CI)
TyG Tertiles				
Tertile 1	44/816 (5.4%)	Reference	Reference	Reference
Tertile 2	58/815 (7.1%)	1.28 (0.84–1.94)	1.27 (0.84–1.93)	1.17 (0.75–1.81)
Tertile 3	101/815 (12.4%)	2.41 (1.65–3.52)	2.38 (1.63–3.47)	1.71 (1.07–2.72)
HOMA-IR Tertiles				
Tertile 1	36/815 (4.4%)	Reference	Reference	Reference
Tertile 2	66/816 (8.1%)	1.62 (1.06–2.49)	1.65 (1.08–2.54)	1.32 (0.84–2.07)
Tertile 3	101/815 (12.4%)	2.77 (1.86–4.12)	2.79 (1.87–4.16)	1.72 (1.07–2.78)
TyG/HOMA-IR				
TyG(−)&HOMA-IR(−)	88/1530 (5.8%)	Reference	Reference	Reference
TyG(+)&HOMA-IR(−)	34/304 (11.2%)	2.09 (1.37–3.21)	2.06 (1.34–3.16)	1.69 (1.04–2.75)
TyG(−)&HOMA-IR(+)	33/305 (10.8%)	1.95 (1.27–2.99)	1.93 (1.26–2.97)	1.39 (0.87–2.24)
TyG(+)&HOMA-IR(+)	48/307 (15.6%)	3.00 (2.05–4.41)	2.98 (2.02–4.38)	1.88 (1.14–3.09)

Model 1: adjusted for age and sex;

Model 2: Model 1 + adjusted for current smoking, current drinking, education and physical activity;

Model 3: Model 2 + adjusted for HbA1c, PP, HDL-cholesterol, LDL-cholesterol, total cholesterol, BMI and medication use of ACEIs or ARBs

PP, pulse pressure; TyG, triglyceride glucose; HOMA-IR, homeostasis model assessment for insulin resistance; OR, odds ratio; CI, confidence interval; BMI, body mass index; HbA1c, glycated hemoglobin; LDL, low density lipoprotein; HDL, high density lipoprotein; ACEIs, angiotensin-converting enzyme inhibitors; ARBs, angiotensin receptor blockers

Similarly, as shown in Supplementary Table 1, those in TyG (+) & HOMA-IR (−) group experienced a greater risk of incident CKD than those in TyG (−) & HOMA-IR (−) group (OR: 1.62, 95% CI 1.01–2.58), and the participants in TyG (+) & HOMA-IR (+) group developed the highest risk (OR: 1.95, 95% CI 1.21–3.12) of incident CKD.

### Stratified analysis for associations between the concordance or discordance groups and new-onset albuminuria and CKD

**Fig. 2:**
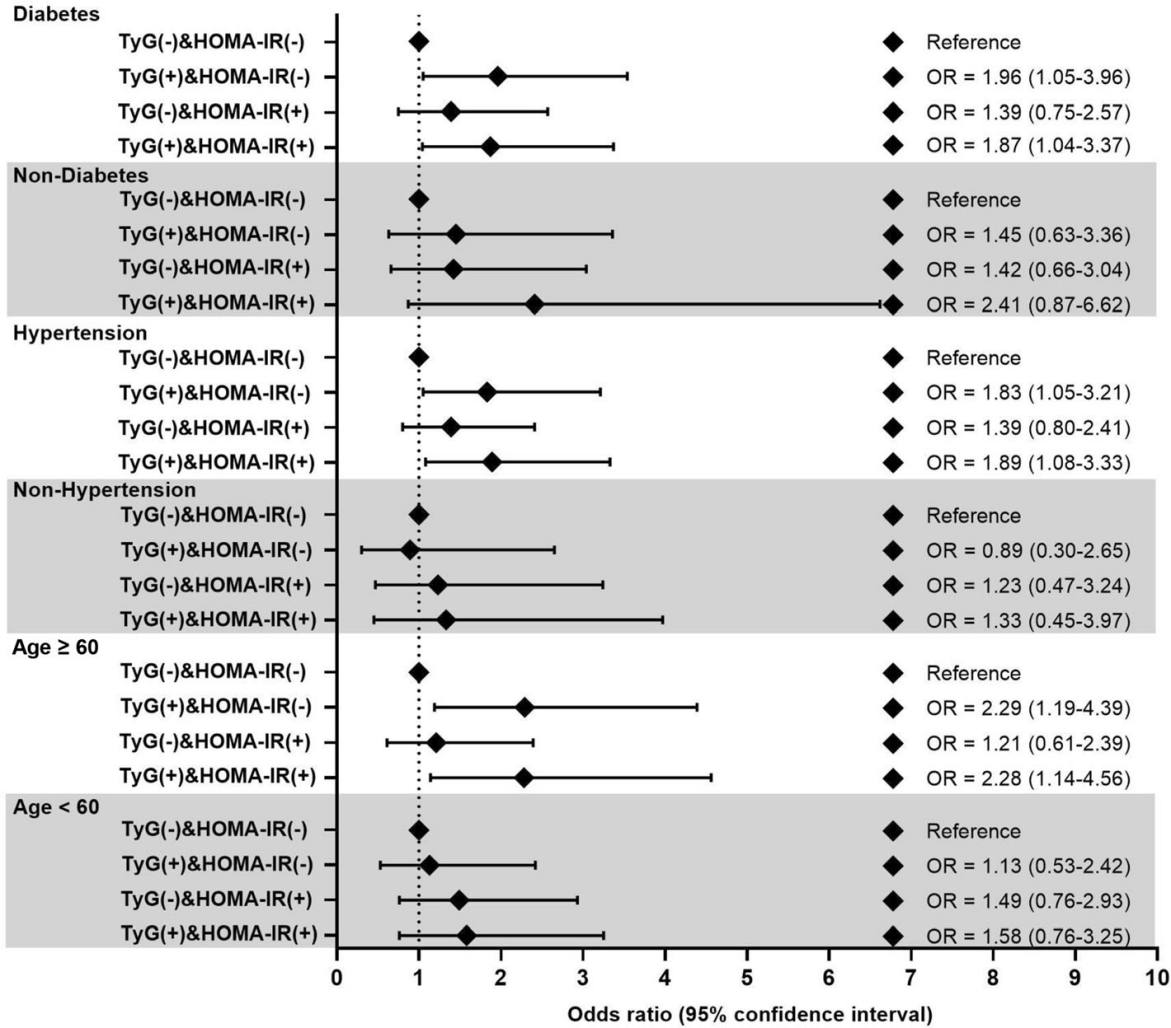
Stratified analysis of the association between concordance or discordance groups of TyG index and HOMA-IR with incident albuminuria. ORs (95% CIs) were adjusted for age, sex, current smoking, current drinking, education, physical activity, HbA1c, PP, HDL-cholesterol, LDL-cholesterol, total cholesterol, BMI and medication treatment of ACEIs or ARBs. TyG, triglyceride glucose; HOMA-IR, homeostasis model assessment for insulin resistance; OR, odds ratio; CI, confidence interval; PP, pulse pressure; BMI, body mass index; HbA1c, glycated hemoglobin; LDL, low density lipoprotein; HDL, high density lipoprotein; ACEIs, angiotensin-converting enzyme inhibitors; ARBs, angiotensin receptor blockers

Subgroup analysis was carried out to explore the association between the concordance or discordance groups and new-onset albuminuria in enrolled participants (Fig. 2). Compared with the TyG (−) & HOMA-IR (−) group, participants in TyG (+) & HOMA-IR (−) group contributed to a significantly higher risk of new-onset albuminuria in patients with diabetes (OR: 1.96, 95% CI 1.05–3.65), with hypertension (OR: 1.83, 95% CI 1.05–3.21) and who were aged>60 (OR: 2.29, 95% CI 1.19–4.39) after multivariable adjustment. The stratified analysis was repeated for the concordance/discordance groups with the outcome of incident CKD and the results were presented in Supplementary Table 2.

## Discussion

This research observed that TyG index was significantly relevant to incident albuminuria in a dose–response manner after adjusting for confounding factors in middle-aged and older participants in China. Furthermore, the discordance analysis showed that participants in TyG (+) & HOMA-IR (−) group experienced a higher risk of incident albuminuria after full adjustment, indicating that the TyG index was more apparently relevant to incident albuminuria than the HOMA-IR. Notably, the risk of incident albuminuria was greatest among the subgroup analyses of individuals with a discordantly high TyG index, suggesting that the TyG index might be a more effective indicator in participants with metabolic abnormalities, such as diabetes, hypertension and ageing. According to what we know, this initial study compared the influence of the TyG index and HOMA-IR on the risk of incident albuminuria in general population firstly.

IR in CKD individuals is closely associated with risk factors resulting in cardiovascular diseases, and the underlying mechanisms may include chronic inflammation, oxidative stress and endothelial dysfunction [21]. The results from previous studies found that patients with early-stage CKD with near-normal creatinine had defects in the insulin-mediated metabolic pathway of glucose. A retrospective cohort research carried out in Korea by Jiang et al. [22] demonstrated that IR was positively associated with the development of albuminuria in healthy individuals without diabetes. Albuminuria is an early manifestation of CKD. In addition, it is also a predictive factor of cardiovascular diseases in subjects with or without diabetes. HIEC is the “gold standard” approach for IR, where as it is not practical to use HIEC in the clinic due to its time-consuming and labour-intensive nature. HOMA-IR is currently the most commonly applied clinical indicator to assess IR. A lot of researches have presented that HOMA-IR is strongly associated with the progression of albuminuria. Recently, the REACTION (Risk Evaluation of Cancers in Chinese Diabetic Individuals: A Longitudinal) study conducted in China [23] found that HOMA-IR was positively related to UACR in prediabetes or diabetes groups, but this relationship was not found in the normal glucose tolerance group. Another large prospective study showed that HOMA-IR quintiles were correlated with the incidence of CKD in adults without diabetes. In the current study, HOMA-IR was further confirmed to have a dose–response relationship with new-onset albuminuria in the general population.

Previous studies have demonstrated a close linkage between the TyG index and cardiovascular diseases. A study including healthy subjects demonstrated that an increasing TyG index was related to a greater risk of cardiovascular disease independent of diabetic status [24]. Limited researches have illustrated the relationship between TyG index and nephric disease. Only one community-based cross-sectional study [14] discovered that higher TyG index was related to elevated microalbuminuria (OR: 1.61, 95% CI 1.22–2.13) and CKD (OR: 1.67, 95% CI 1.10–2.50) risk. This finding was consistent with the present study. Furthermore, data from this study supported that the TyG index might be an improved IR surrogate marker compared with HOMA-IR in the early stage of renal disease. According to the present study, participants in TyG (+) & HOMA-IR (−) group experienced a significantly greater risk of incident albuminuria independent of traditional cardiovascular disease risk factors in discordant analysis, first elaborating the diagnostic value of the TyG index in the early stage of nephrotic damage.

Abnormal metabolic status such as diabetes and hypertension are established risk factors of cardiovascular diseases. Previous studies [25–27] have illustrated that diabetes and hypertension harmed the microvascular system, and the underlying mechanism between diabetes, hypertension and microcirculation might include hypertrophic remodelling in small vessels, endothelial dysfunction and vascular dysfunction at the capillary network. These ultimately lead to an increase in microvascular permeability to large molecules (such as albumin) and impaired insulin sensitivity. Since then, several epidemiological studies [28, 29] have reported the UACR as a predictive index of cardiovascular events and mortality in diabetes, hypertension and the general population. In the current study, the risk of incident albuminuria across the 4 concordance or discordance groups in different cardiovascular metabolic disorder groups was further investigated, showing that the TyG index performed more effective than HOMA-IR in identifying incident albuminuria risk in subjects with higher cardiovascular metabolic risk, such as diabetes status and hypertension status.

Ageing is related to IR to a certain content. A community-based study of participants aged ≥ 65 years old in northern Shanghai [14] obtained a result that elevated TyG index had a greater risk of new-onset microalbuminuria or CKD. Rather than comparing the TyG index with other markers of IR in ageing participants, they revealed an linkage between the TyG index and nephric dysfunction. In this research, through the discordance analysis with HOMA-IR, the results further proved that the TyG index could recognize early renal stage in elderly individuals, which could improve the early detection of diseases.

### Comparisons with other studies and what does the current work add to the existing knowledge

Previous studies have demonstrated the associations between the two indexes mentioned above with microalbuminuria or CKD separately in specific populations, such as elderly individuals or those with diabetes [14, 23]. However, this study compared the performance of TyG index with HOMA-IR in general population. Meanwhile, TyG index implemented more effective than HOMA-IR at identifying new-onset albuminuria in subjects with metabolic disorders.

### Study strengths and limitations

This current study has some strengths. This study directly compared the effectiveness of the TyG index and HOMA-IR at evaluating new-onset albuminuria risk in the general population for the first time and approved that the TyG index was better at identifying metabolic disorder subjects with new-onset albuminuria than HOMA-IR. There are also several limitations of this study that should be considered. First, this research is presented in a Chinese population aged more than 40 years old, which is not generalizable to other ethnic groups. Second, the length of the follow-up time (3.9 years follow-up) may not have been sufficient to completely capture the probable occurrence of albuminuria and CKD. Third, the widespread use of the TyG index as the best cut-off value as an alternative marker requires further research in future studies. Lastly, some underlying metabolic-related disorder factors that might influence the results were not considered in the current research, like primary triglyceride abnormalities.

## Conclusion

This present study summarized that participants companying a discordantly high TyG index had a significantly greater incident albuminuria risk, especially in subjects with cardiovascular metabolic abnormalities. This conclusion supports the clinical value of the TyG index, with its readily available and reliable feature in clinical practice and could help clinicians identify the early stage of CKD in advance.

## Supplementary Information

**Additional file 1.** Supplementary Table 1 Incidence of CKD using the TyG index, HOMA-IR and concordance/discordance groups (N=2448). Supplementary Table 2 Stratified analyses of the association between concordance/discordance groups and CKD. Supplementary Figure 1 Participant Flow Diagram of CKD outcome. Supplementary Figure 2 Scatterplots and prevalence of discordance and concordance defined according to the upper quartile values of TyG index and HOMA-IR.

## Data Availability

The datasets generated during and/or analysed during the current study are not publicly available due to the data privacy policy of the cohort but are available from the corresponding author on reasonable request.

## Abbreviations

ACEI: angiotensin-converting enzyme inhibitor
ADA: American Diabetes Association
ARB: angiotensin receptor blocker
BMI: body mass index
BP: blood pressure
CI: confidence interval
CKD: chronic kidney disease
DBP: diastolic blood pressure
eGFR: estimated glomerular filtration rate
FPG: fasting plasma glucose
HbA1c: glycated haemoglobin
HDL: high-density lipoprotein
HIEC: hyperinsulinaemic-euglycaemic clamp
HOMA-IR: homeostasis model assessment of insulin resistance
IR: insulin resistance
LDL: low-density lipoprotein
PP: pulse pressure
OGTT: oral glucose tolerance test
OR: odds ratio
REACTION Study: Risk Evaluation of Cancers in Chinese Diabetic Individuals: A Longitudinal Study
SBP: systolic blood pressure
TG: triglyceride
TyG: triglyceride-glucose
UACR: urinary albumin-to-creatinine ratio
