# Supplementary material for: Discordance between the triglyceride glucose index and HOMA-IR in incident albuminuria: a cohort study from China
Source: Lipids Health Dis. 2021 Dec 5;20:176. doi: 10.1186/s12944-021-01602-w (PMC8647334; doi:10.1186/s12944-021-01602-w)

Supplementary Table 1 Incidence of CKD using the TyG index, HOMA-IR and concordance/discordance groups (N=2448).

|  |  | CKD | | |
| --- | --- | --- | --- | --- |
|  |  | Model 1 | Model 2 | Model 3 |
|  | Cases/participants | OR (95% CI) | OR (95% CI) | OR (95% CI) |
| TyG Tertiles | | | | |
| Tertile 1 | 49/816 | Reference | Reference | Reference |
| Tertile 2 | 66/816 | 1.29 (0.87-1.92) | 1.28 (0.86-1.90) | 1.19 (0.78-1.80) |
| Tertile 3 | 115/816 | 2.46 (1.72-3.52) | 2.44 (1.70-3.50) | 1.88 (1.21-2.92) |
| HOMA-IR Tertiles | | | | |
| Tertile 1 | 42/816 | Reference | Reference | Reference |
| Tertile 2 | 71/816 | 1.49 (0.99-2.23) | 1.52 (1.01-2.27) | 1.22 (0.80-1.88) |
| Tertile 3 | 117/816 | 2.74 (1.89-3.97) | 2.78 (1.91-4.04) | 1.78 (1.14-2.80) |
| TyG/HOMA-IR | | | | |
| TyG(-)&HOMA-IR(-) | 101/1531 | Reference | Reference | Reference |
| TyG(+)&HOMA-IR(-) | 36/305 | 1.90 (1.26-2.87) | 1.88 (1.24-2.84) | 1.62 (1.01-2.58) |
| TyG(-)&HOMA-IR(+) | 39/305 | 1.98 (1.33-2.96) | 1.98 (1.32-2.95) | 1.45 (0.93-2.26) |
| TyG(+)&HOMA-IR(+) | 54/307 | 2.96 (2.05-4.27) | 2.96 (2.05-4.28) | 1.95 (1.21-3.12) |

Model 1: adjusted for age and sex;

Model 2: Model 1 + adjusted for smoking status, drinking status, education and physical activity;

Model 3: Model 2 + adjusted for HbA1c, PP, HDL-cholesterol, LDL-cholesterol, total cholesterol, BMI and medication use of ACEIs or ARBs.

PP, pulse pressure; TyG, triglyceride glucose; HOMA-IR, homeostasis model assessment for insulin resistance; CKD, chronic kidney disease; OR, odds ratio; CI, confidence interval; BMI, body mass index; HbA1c, glycated hemoglobin; LDL, low density lipoprotein; HDL, high density lipoprotein; ACEIs, angiotensin-converting enzyme inhibitors; ARBs, angiotensin receptor blockers.

Supplementary Table 2 Stratified analyses of the association between concordance/discordance groups and CKD.

|  |  | CKD | | |
| --- | --- | --- | --- | --- |
|  |  | Model 1 | Model 2 | Model 3 |
|  | Cases/participants | OR (95% CI) | OR (95% CI) | OR (95% CI) |
| Diabetes | | | | |
| TyG(-)&HOMA-IR(-) | 37/330 | Reference | Reference | Reference |
| TyG(+)&HOMA-IR(-) | 25/142 | 1.82 (1.03-3.22) | 1.79 (1.01-3.17) | 1.83 (0.99-3.38) |
| TyG(-)&HOMA-IR(+) | 28/167 | 1.79 (1.04-3.09) | 1.76 (1.01-3.05) | 1.82 (1.03-3.22) |
| TyG(+)&HOMA-IR(+) | 46/246 | 2.09 (1.28-3.41) | 2.18 (1.33-3.57) | 2.13 (1.21-3.73) |
| Non-Diabetes | | | | |
| TyG(-)&HOMA-IR(-) | 65/1227 | Reference | Reference | Reference |
| TyG(+)&HOMA-IR(-) | 12/173 | 1.45 (0.76-2.78) | 1.42 (0.74-2.73) | 1.59 (0.74-3.43) |
| TyG(-)&HOMA-IR(+) | 11/139 | 1.44 (0.73-2.83) | 1.40 (0.71-2.77) | 1.30 (0.63-2.69) |
| TyG(+)&HOMA-IR(+) | 8/63 | 2.69 (1.21-6.00) | 2.59 (1.16-5.80) | 2.60 (1.01-6.73) |
| Hypertension | | | | |
| TyG(-)&HOMA-IR(-) | 63/709 | Reference | Reference | Reference |
| TyG(+)&HOMA-IR(-) | 31/188 | 2.20 (1.36-3.55) | 2.20 (1.36-3.55) | 1.82 (1.06-3.11) |
| TyG(-)&HOMA-IR(+) | 31/204 | 1.98 (1.24-3.19) | 2.01 (1.25-3.23) | 1.48 (0.88-2.48) |
| TyG(+)&HOMA-IR(+) | 45/223 | 2.83 (1.84-4.36) | 2.89 (1.87-4.47) | 1.99 (1.16-3.42) |
| Non-Hypertension | | | | |
| TyG(-)&HOMA-IR(-) | 45/857 | Reference | Reference | Reference |
| TyG(+)&HOMA-IR(-) | 6/119 | 0.93 (0.36-2.43) | 0.90 (0.35-2.37) | 0.84 (0.29-2.42) |
| TyG(-)&HOMA-IR(+) | 8/106 | 1.54 (0.69-3.42) | 1.56 (0.70-3.49) | 1.36 (0.57-3.24) |
| TyG(+)&HOMA-IR(+) | 9/85 | 2.30 (1.07-4.96) | 2.18 (1.00-4.75) | 1.69 (0.64-4.46) |
| Age≥60 | | | | |
| TyG(-)&HOMA-IR(-) | 52/565 | Reference | Reference | Reference |
| TyG(+)&HOMA-IR(-) | 22/131 | 2.12 (1.23-3.67) | 2.15 (1.24-3.73) | 2.10 (1.14-3.87) |
| TyG(-)&HOMA-IR(+) | 20/150 | 1.52 (0.87-2.66) | 1.55 (0.89-2.73) | 1.30 (0.71-2.39) |
| TyG(+)&HOMA-IR(+) | 32/146 | 3.01 (1.82-4.96) | 3.14 (1.90-5.21) | 2.36 (1.25-4.47) |
| Age＜60 | | | | |
| TyG(-)&HOMA-IR(-) | 49/966 | Reference | Reference | Reference |
| TyG(+)&HOMA-IR(-) | 14/174 | 1.73 (0.90-3.30) | 1.60 (0.83-3.08) | 1.07 (0.50-2.27) |
| TyG(-)&HOMA-IR(+) | 19/155 | 2.88 (1.63-5.09) | 2.76 (1.55-4.92) | 1.55 (0.80-2.99) |
| TyG(+)&HOMA-IR(+) | 22/161 | 3.15 (1.81-5.47) | 2.98 (1.70-5.21) | 1.50 (0.73-3.06) |
| Male | | | | |
| TyG(-)&HOMA-IR(-) | 33/599 | Reference | Reference | Reference |
| TyG(+)&HOMA-IR(-) | 18/148 | 2.66 (1.43-4.93) | 2.72 (1.46-5.06) | 2.17 (1.05-4.46) |
| TyG(-)&HOMA-IR(+) | 15/102 | 2.99 (1.54-5.79) | 3.09 (1.59-6.04) | 2.13 (1.00-4.56) |
| TyG(+)&HOMA-IR(+) | 13/119 | 2.42 (1.22-4.82) | 2.67 (1.33-5.37) | 0.69 (0.24-1.96) |
| Female | | | | |
| TyG(-)&HOMA-IR(-) | 68/932 | Reference | Reference | Reference |
| TyG(+)&HOMA-IR(-) | 18/157 | 2.48 (0.84-2.61) | 1.47 (0.83-2.61) | 1.26 (0.66-2.40) |
| TyG(-)&HOMA-IR(+) | 24/203 | 1.61 (0.97-2.66) | 1.59 (0.96-2.63) | 1.23 (0.72-2.12) |
| TyG(+)&HOMA-IR(+) | 41/188 | 3.22 (2.08-4.99) | 3.09 (1.99-4.81) | 1.81 (1.01-3.27) |

Model 1: adjusted for age and sex;

Model 2: Model 1 + adjusted for smoking status, drinking status, education and physical activity;

Model 3: Model 2 + adjusted for HbA1c, PP, HDL-cholesterol, LDL-cholesterol, total cholesterol, BMI and medication use of ACEIs or ARBs.

PP, pulse pressure; TyG, triglyceride glucose; HOMA-IR, homeostasis model assessment for insulin resistance; CKD, chronic kidney disease; OR, odds ratio; CI, confidence interval; BMI, body mass index; HbA1c, glycated hemoglobin; LDL, low density lipoprotein; HDL, high density lipoprotein; ACEIs, angiotensin-converting enzyme inhibitors; ARBs, angiotensin receptor blockers.

Supplementary Figure 1 Participant Flow Diagram of CKD outcome. TyG, triglyceride glucose; HOMA-IR, homeostasis model assessment for insulin resistance; CKD, chronic kidney disease; UACR, urinary albumin-to-creatinine ratio; eGFR: estimated glomerular filtration rate.


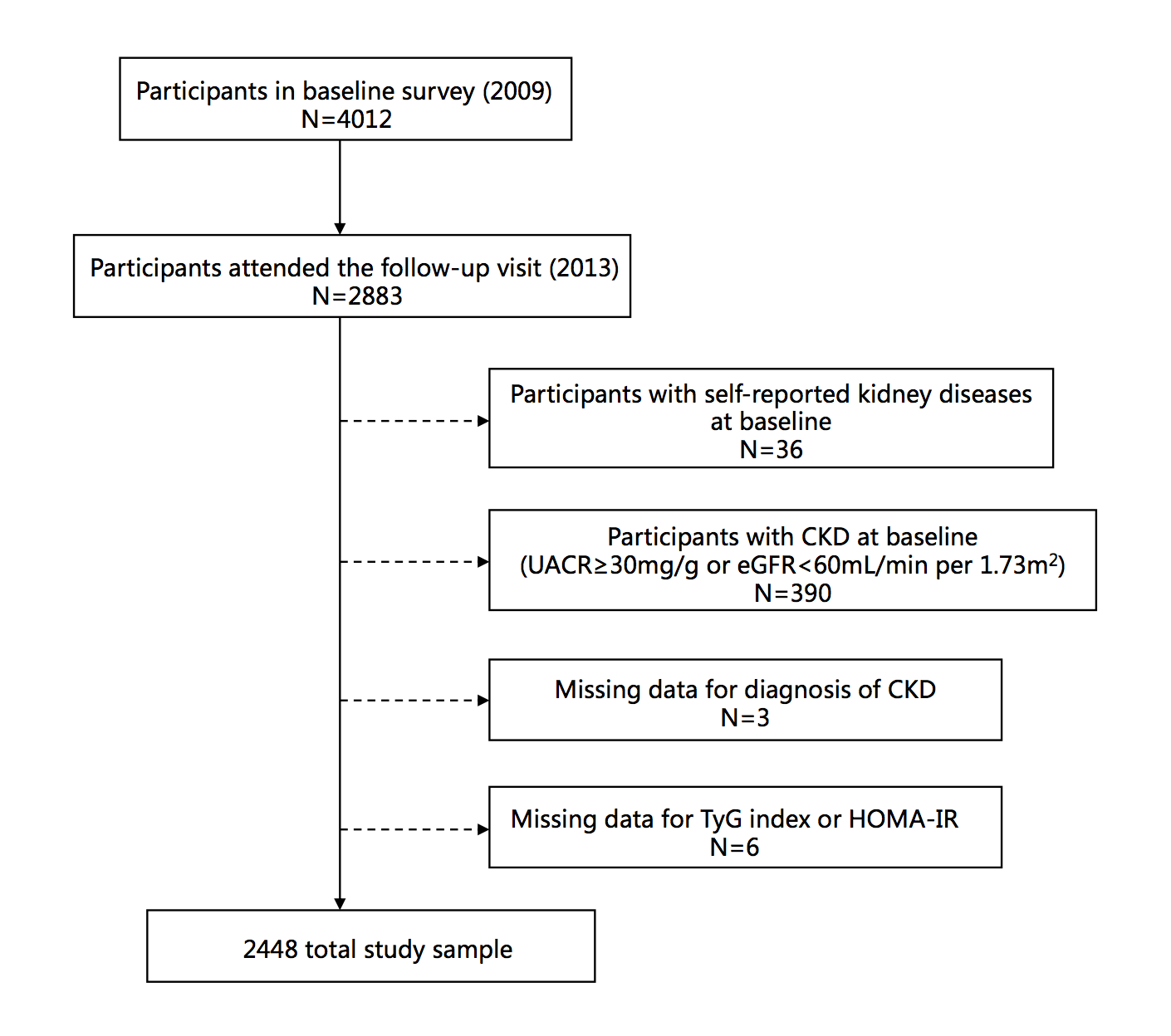


Supplementary Figure 2 Scatterplots and prevalence of discordance and concordance defined according to the upper quartile values of TyG index and HOMA-IR. TyG, triglyceride glucose; HOMA-IR, homeostasis model assessment for insulin resistance;


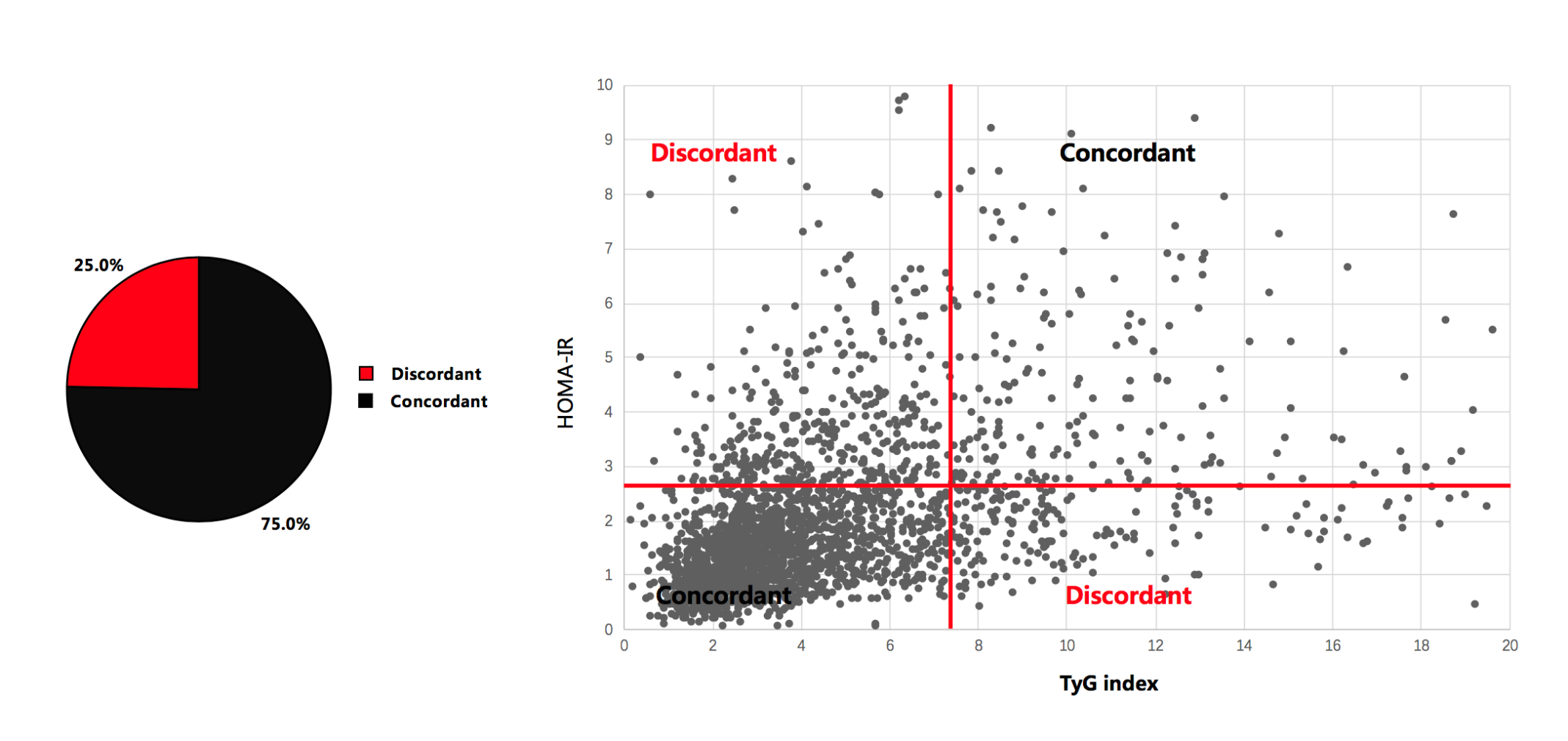

Supplement: Supplementary file 1 — Additional file 1. Supplementary Table 1 Incidence of CKD using the TyG index, HOMA-IR and concordance/discordance groups (N=2448). Supplementary Table 2 Stratified analyses of the association between concordance/discordance groups and CKD. Supplementary Figure 1 Participant Flow Diagram of CKD outcome. Supplementary Figure 2 Scatterplots and prevalence of discordance and concordance defined according to the upper quartile values of TyG index and HOMA-IR. [file 12944_2021_1602_MOESM1_ESM.docx]
